# Interaction of microtubule depolymerizing agent indanocine with different human αβ tubulin isotypes

**DOI:** 10.1371/journal.pone.0194934

**Published:** 2018-03-27

**Authors:** Bajarang Vasant Kumbhar, Dulal Panda, Ambarish Kunwar

**Affiliations:** Department of Biosciences and Bioengineering, Indian Institute of Technology Bombay, Powai, Mumbai, Maharashtra, India; Weizmann Institute of Science, ISRAEL

## Abstract

Tubulin isotypes are known to regulate the stability and dynamics of microtubules, and are also involved in the development of resistance against microtubule-targeted cancer drugs. Indanocine, a potent microtubule depolymerizing agent, is highly active against multidrug-resistant (MDR) cancer cells without affecting normal cells. It is known to disrupt microtubule dynamics in cells and induce apoptotic cell death. Indanocine is reported to bind to tubulin at the colchicine site i.e. at the interface of αβ tubulin heterodimer. However, it’s precise binding mode, involved molecular interactions and the binding affinities with different αβ-tubulin isotypes present in MDR cells are not well understood. Here, the binding affinities of human αβ-tubulin isotypes with indanocine were examined, employing the molecular modeling approach i.e. docking, molecular dynamics simulation and binding energy calculations. Multiple sequence analysis suggests that the amino acid sequences are different in the indanocine binding pockets of βI, βIIa, βIII and βVI isotypes. However, such differences are not observed in the amino acid sequences of βIVa, βIVb, and βV tubulin isotypes at indanocine binding pockets. Docking and molecular dynamics simulation results show that indanocine prefers the interface binding pocket of αβIIa, αβIII, αβIVb, αβV, and αβVI tubulin isotypes; whereas it is expelled from the interface binding pocket of αβIVa and αβI-tubulin isotypes. Further, binding free energy calculations show that αβVI has the highest binding affinity and αβI has the lowest binding affinity for indanocine among all β-tubulin isotypes. The binding free energy decreases in the order of αβVI > αβIVb > αβIIa > αβIII > αβV > αβIVa > αβI. Thus, our study provides a significant understanding of involved molecular interactions of indanocine with tubulin isotypes, which may help to design potent indanocine analogues for specific tubulin isotypes in MDR cells in future.

## Introduction

Microtubules are dynamic cytoskeleton filamentous proteins; they play essential roles in cell division, cell movement, and intracellular transport [1]. They are polymers of α/β-tubulin heterodimers. These α/β-tubulin are encoded by multiple genes which are expressed tissue-specifically e.g. βI is ubiquitous, βIII is expressed in neuronal and testicular cells, βIVa in neuronal and glial cells, βVI is observed in the erythroid cells and platelets etc. [2,3]. In humans, ten β-tubulin and seven α-tubulin isotypes exist [4,5] and these isotypes show considerable difference at the C-terminal end. Tubulin isotypes composition plays an essential role in regulating microtubule dynamics [6–8]. The essential roles of microtubules during the cell division make them important and attractive targets to design new anticancer agents. Anticancer agents are generally classified into microtubule stabilizing agents (MSA) and microtubule destabilizing agents (MDA). The MSAs prefer to bind at the ‘taxol site’ (e.g. paclitaxel, epothilone) whereas MDAs prefer to bind at the ‘colchicine’ and ‘vinca’ site (e.g. colchicine, indanocine, vinblastine), leading to cell death due to apoptosis in both the cases [5].

A major difficulty with the effectiveness of microtubule-targeting agents arises from the emergence of drug resistance, which is mainly due to the mutation in β-tubulin protein and an increased expression of the P-glycoprotein pump [9]. In addition, an over-expression of β-tubulin isotypes in cancerous cells also plays a crucial role in drug resistance, as they show lesser binding affinities for numerous anti-mitotic agents [10–13]. Different tubulin isotypes are overexpressed in cancerous cells; particularly overexpression βI, βII, βIII, βIV, and βV-tubulin isotypes are associated with multidrug-resistant cancer [14–17]. Furthermore, it has also been observed that βII, βIII and βIV tubulin isotypes show differential binding affinities for a variety of anticancer drugs e.g. taxol, colchicine, DAMA-colchicine, and nocodazole [10,12,13,18]. Therefore, these drug-resistant tubulin isotypes have been highlighted as interesting targets for designing new anticancer agents.

**Fig 1:**
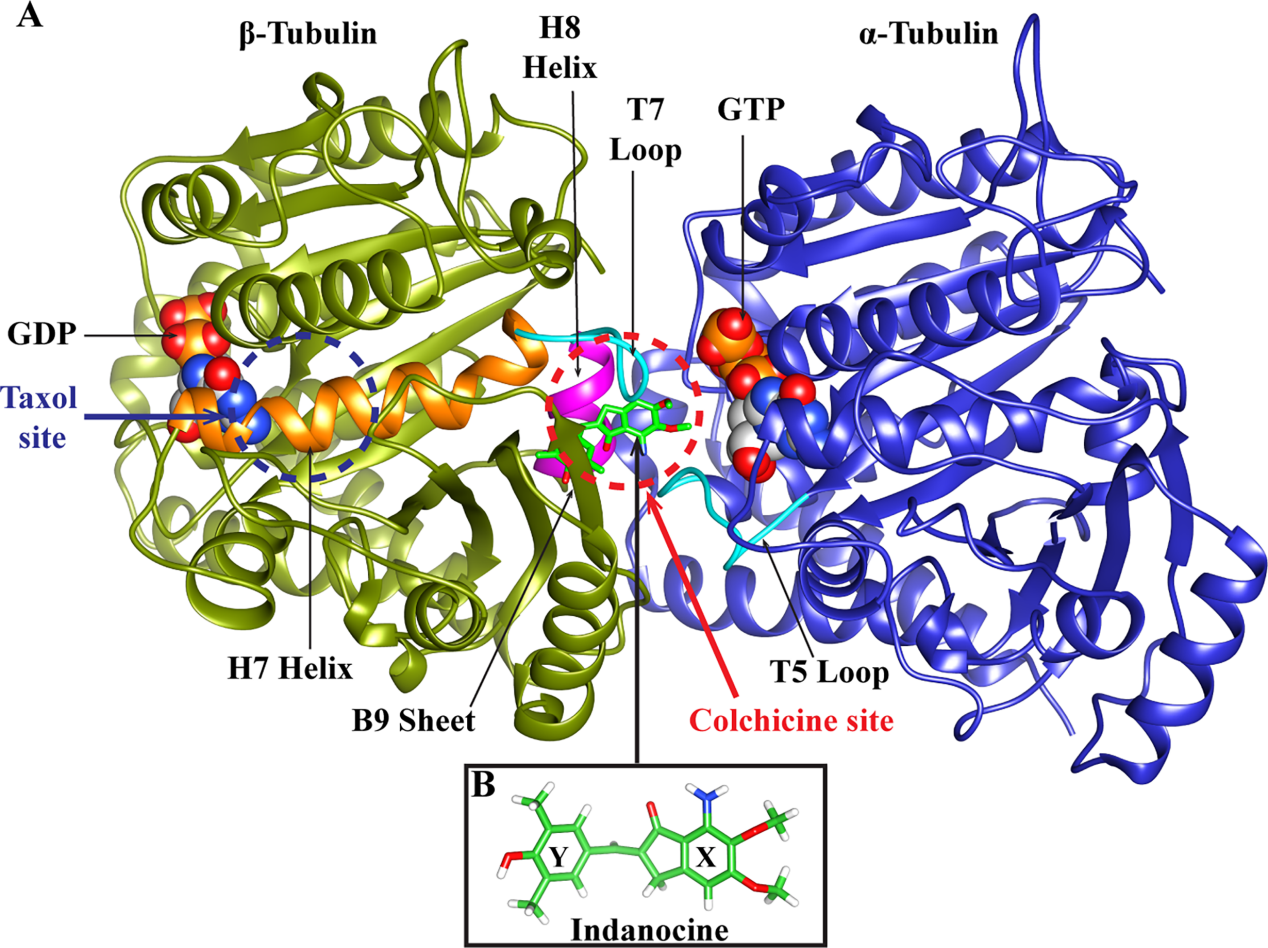
The representative structure of αβ-tubulin dimer and indanocine. (A) α/β-tubulin subunits. α subunit is shown in blue color and β subunit is shown in olive green color. Indanocine binding site i.e. ‘colchicine site’ is at the interface of α-tubulin and β-tubulin heterodimer (red dotted circle); while the ‘taxol site’ is present on only β-tubulin over the H7 helix (blue dotted circle). The indanocine binding pocket consists of H7 helix (shown in orange), T7 loop (shown in cyan), H8 helix (shown in magenta) and T5 loop of α-tubulin (shown in cyan). Here, H7 denotes the α-helix number 7, T7 stands for T loop number 7, and B9 implies the β-sheet number 9. The GTP in α-tubulin, GDP in β-tubulin are shown using space-fill models. The white, grey, red, blue and golden yellow colors represent carbon, hydrogen, oxygen, nitrogen and phosphorous atoms, respectively. (B) The structure of indanocine has a dimethoxyaniline group (labeled as X ring) and a dimethylphenol group (labeled as Y ring). The carbon, oxygen, nitrogen, and hydrogen atoms of indanocine are shown in green, red, blue and white color, respectively.

Indanocine, a synthetic indanone, is a microtubule depolymerizing agent with potent anti-proliferative activity [19]. Indanocine acts against multidrug-resistant (MDR) cancer cells and kills non-dividing and quiescent cells [19], but it does not affect the normal non-proliferating cells. Indanocine affects the microtubule dynamicity at very low concentration and inhibits the migration of metastatic cancer cells [20]. It prefers to bind at the interface of αβ tubulin heterodimer i.e. at the colchicine binding site [21]. Indanocine is a flexible molecule in which the indanone group and the dimethylphenol group are connected by a single bond (Fig 1B).

Indanocine binds to the αβ tubulin heterodimer in a reversible manner, and it binds to tubulin at a faster rate than colchicine [21]. Indanocine is a potent microtubule de-polymerizing agent and it acts against multidrug-resistant (MDR) cancer cells without affecting the normal cells. However, its precise binding mode involved molecular interactions and the binding affinities with different αβ-tubulin isotypes present in MDR cells are not well understood. Here, the binding affinities of human αβ-tubulin isotypes with indanocine were examined, employing molecular modeling approach.

## Computational methodology

### Sequence analysis of β tubulin isotypes

The amino acid sequences of seven different human β-tubulin isotypes were taken from the UniProt database. The UniProt IDs of these β-tubulin sequences are as follows βI(Q9H4B7), βIIa(Q13885), βIII(Q13509), βIVa(P04350), βIVb(P68371), βV(P07437), and βVI(Q9BUF5). To the best of our knowledge, structures of human tubulin isotypes bound with indanocine have not been determined using either X-Ray Crystallography or NMR techniques. Hence, the crystal structure of αβ-tubulin heterodimer (1SA0.pdb) from Protein Database was used as a template or reference. Bovine 1SA0.pdb has the βII tubulin which is identical in sequence to human βII tubulin [10]. Therefore, crystal structure 1SA0.pdb was used as a template to build the 3D model of human tubulin isotypes. The sequence alignments of seven different human β-tubulin isotypes and the template sequence were performed to pinpoint the difference in residues at the indanocine binding pocket using Clustal Omega tool of EMBL-EBI [22].

### Homology modeling of human αβ-tubulin isotypes

In this study, the colchicine-bound crystal structure of αβ-tubulin heterodimer (source code:1SA0.pdb) [23] was used as the template structure to build the 3D structures of seven human tubulin isotypes using homology modeling technique. Here, word template implies an initial structure used to build the desired model structure in the absence of required crystal structure, using homology modeling approach. The crystal structure of αβ-tubulin heterodimer ‘1SA0.pdb’ is from bovine source with a resolution of 3.58Å [23]. Previously, we have shown that human βIIa, βIII, βIVa are identical i.e. 100% similar to bovine βIIa, βIII, and βIVa [10]. Similarly, we have also checked the similarity between other tubulin isotypes, and have found that the human βIVb, βV, and βVI are also identical i.e. 100% similar to bovine βIVb (Q3MHM5), βV(Q2KJD0), βVI(Q2HJ81) whereas human βI and bovine βI (E1BJK2) show 88.89% similarity.

We considered chains A and B from the crystal structure 1SA0.pdb for homology modeling whose missing residues of β-tubulin (37 to 47) and α-tubulin (1, 275–284) were built using MODELLER9v18 [24]. This refined structure of αβ-tubulin heterodimers obtained from MODELLER9v18 was used as template and will be referred to as tubulin 1SA0 hereafter. The homology models of seven human αβ-tubulin isotype heterodimers such as αβI, αβIIa, αβIII, αβIVa, αβIVb, αβV, and αβVI were built using the template structure 1SA0.pdb through MODELLER9v18 and the best models were selected on the basis of their DOPE score [24]. The C-terminal ends of tubulin isotypes were not included in our study as they are not present at the interface of αβ-tubulin. Therefore, we do not expect C-terminal tails to play a direct role in drug binding at the interface. C-terminal ends of the different isotypes have been modeled by Luchko et al. [25] for conformational analysis. A recent study of interaction of different human tubulin isotypes with drug DAMA-Colchicine [10] shows that C-terminal end does not qualitatively affect the binding of DAMA-Colchicine with tubulin isotypes. The stereochemical quality of αβ-tubulin models was evaluated using PROCHECK [26] and Verify-3D [27] to ensure the reliability of the homology models, whose details are given in the Supplementary S1 Text. Subsequent energy minimization was performed on tubulin 1SA0 and seven different αβ-tubulin isotype heterodimers using 5,000 steps of steepest descent method; out of which 3,000 steps were of conjugate gradient method using AMBER12 software [28]. For energy minimization, the parameters of guanosine triphosphate (GTP), and guanosine diphosphate (GDP) and Mg^2+^ were obtained from the AMBER database [29,30]. These energy minimized structures of αβ-tubulin isotypes were then used for the docking of indanocine using AutoDock4.2 [31].

In this study, we kept the α-tubulin as constant and varied the beta-tubulin isotypes as accurate combinations of different αβ-tubulin isotypes is not well known experimentally. Indanocine works on various multi-drug resistant cancer cell types where over-expression of different beta-tubulin isotypes (as compared to the α-tubulin isotypes) leads to drug-resistance. Hence, we did not use all possible combinations of different αβ-tubulin isotypes in this study.

### Molecular docking of indanocine with αβ-tubulin isotypes

To identify the interactions of tubulin 1SA0 and different human αβ-tubulin isotypes with indanocine, molecular docking was performed using AutoDock4.2 [31]. For molecular docking, energy-minimized 3D atomic coordinates of indanocine were generated using the PRODRG server [32]. Since indanocine was suggested to bind at the interface of αβ tubulin [21], an autogrid was used to outline the putative binding pocket around the interface of αβ tubulin [31]. The Gasteiger charges were added to αβ tubulin using AutoDock4.2 [31]. Here, we used local docking methodology to delineate the binding mode of indanocine with tubulin [33,34].

A grid box of size 60Å×60Å×60Å with a spacing of 0.375Å was prepared at the αβ tubulin interface i.e. the putative indanocine binding site. The Lamarckian Genetic Algorithm (LGA) was used for molecular docking with default parameters [31]. Here, a total of 50 independent flexible ligand dockings were conducted, each composed of 100 LGA runs, which yielded a total of 5,000 conformations. They were subsequently clustered using an all-atom RMSD cut-off of 4Å; which were then analyzed considering cluster size and binding free energy calculated by a scoring function of AutoDock4.2 [31,35]. The lowest binding free energy docked conformation of indanocine was selected for further hydrogen bonding interactions analysis and for molecular dynamics simulations.

### Molecular dynamics simulation

Molecular dynamics simulations were performed for indanocine-docked complexes with tubulin 1SA0 and seven different human αβ tubulin isotypes i.e. αβI, αβIIa, αβIII, αβIVa, αβIVb, αβV, αβVI, and αβVII using the SANDER module of AMBER12 [28]. The AMBER ff99SB force field was applied for protein, and the parameters for guanosine triphosphate (GTP), guanosine diphosphate (GDP) and Mg^2+^ were taken from the AMBER database [29,30]. Parameters for indanocine were generated by using the ‘Antechamber’ module of AMBER12 [10,36]. The implicit ‘Generalized Born/Surface Area (GB/SA)’ model was used to represent the solvent effect by using the parameters described by Tsui [37] to explore the interactions of protein-ligand [10,36]. The molecular dynamics simulations steps such as minimization, heating, equilibration and production run were performed using the same parameters as in our earlier studies [10]. The trajectories of molecular dynamics simulations were visualized and analyzed using VMD [38] and PyMol [39]. VMD was employed to produce the molecular dynamics simulation movies by setting the Trajectory Smoothing Window size for protein to 5, and for GTP, GDP and indanocine to 3.

### Binding energy calculations

The αβ tubulin isotype-indanocine binding free energy calculations were estimated using MM-GBSA approaches using AMBER12 [28]. The binding free energy was calculated using 10,000 frames from the last 2ns of molecular dynamics trajectories with an interval of 5 for each system using mmpbsa module of AMBER12 similar to our earlier study [10]. The entropy calculations are computationally expensive and hence omitted in this study, as done in an earlier study [10]. The need of explicit calculation of the entropy can be avoided in this study as we are comparing the relative trend of binding free energies of different isotypes which are related systems (there is a difference of few residues among them) [10,40].

## Results and discussion

### Sequence analysis and homology modeling of αβ-tubulin isotypes

**Fig 2:**
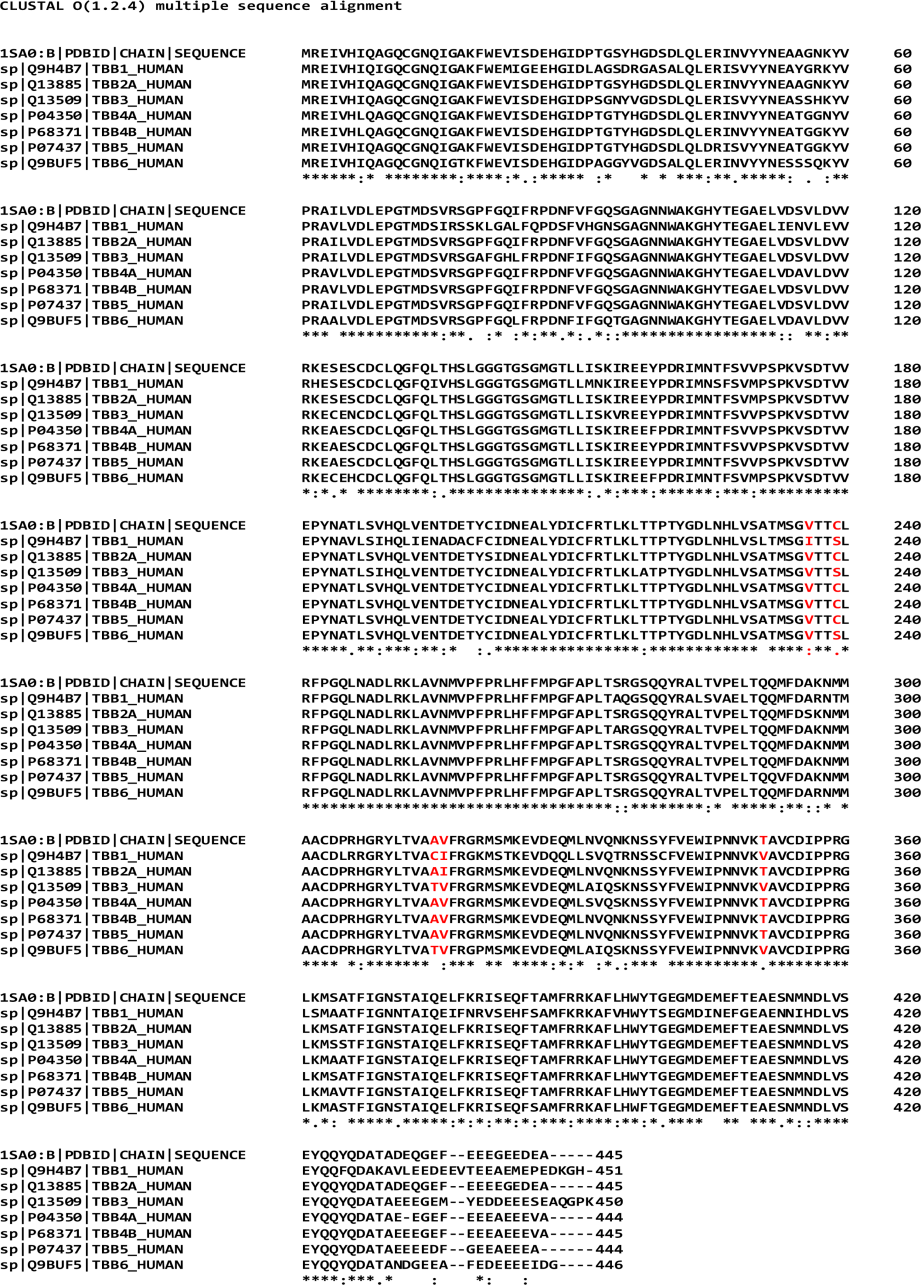
Multiple sequence alignment of human β-tubulin isotypes. The isotype βI shows variations of residues Val236-Ile, Cys239-Ser, Ala315-Cys, Val316-Ile and Thr351-Val, βIIa shows a change of Val316-Ile, βIII and βVI shows a change of Cys239-Ser, Ala315-Thr, and Thr351-Val at the indanocine binding pocket. Residue variations in the indanocine binding pocket are shown in red. Symbol ‘ * ‘ denotes positions of amino acid which have a single, fully conserved amino acid residue; the symbol ‘: ‘ denotes conservation between groups of strongly similar properties of amino acid; the symbol ‘.’ denotes the conservation between groups of weakly similar properties of amino acids, and the symbol ‘—‘ denotes gaps inserted to maximize sequence alignment [22].

Multiple sequence analysis of the seven above-mentioned different human β-tubulin isotypes against bovine β_II_ tubulin (PDB code: 1SA0, chain B) as reference sequence was performed using Clustal Omega tools of EMBL-EBI [22]. The multiple sequence analysis study shows that human β-tubulin isotypes show residue composition variations at different locations (Fig 2). We further analyzed the residue composition variations at the indanocine binding pocket of different β-tubulin isotypes. The indanocine binding pocket of βI has five residue changes i.e. Val236-Ile, Cys239-Ser, Ala315-Cys, Val316-Ile, and Thr351-Val, βIIa has a single amino acid change i.e. Val316-Ile, βIII has three residue changes i.e. Cys239-Ser, Ala315-Thr, and Thr351-Val (Fig 2), and βVI also has three residue changes similar to βIII tubulin isotypes such as Cys239-Ser, Ala315-Thr, and Thr351-Val. There is no residue composition variation in βIVa, βIVb, and βV at the indanocine binding pocket (Fig 2). We then built homology models of these seven human αβ-tubulin isotypes and performed molecular docking of indanocine and molecular dynamics simulations of αβ tubulin-indanocine complexes to explore the effect of residue composition on the binding interaction of indanocine.

### Molecular docking of indanocine with αβ-tubulin isotypes

**Fig 3:**
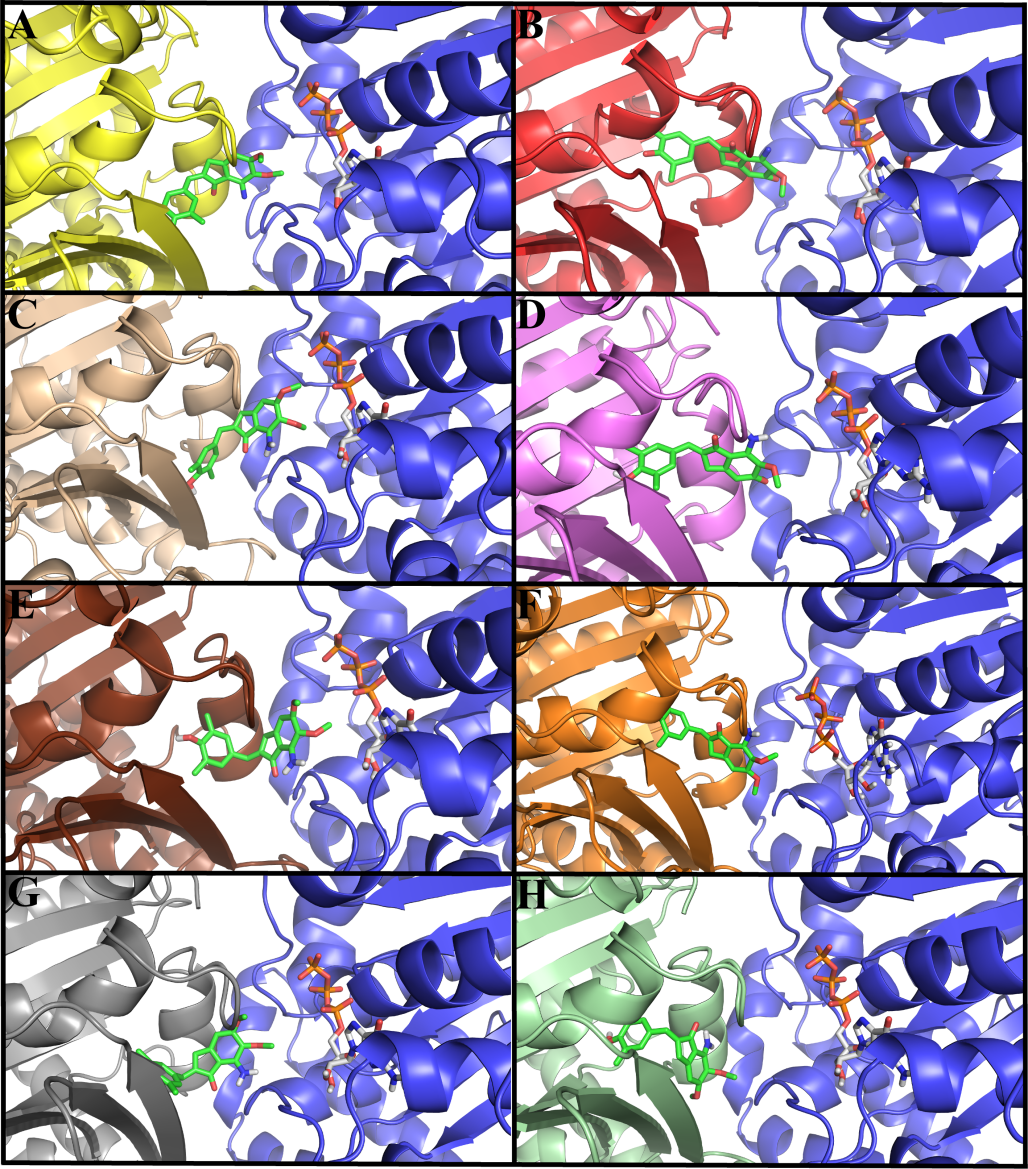
Docked conformations of indanocine with αβ-tubulin isotypes. The color for α-tubulin is blue in all αβ-tubulin heterodimers while color of different β-tubulin isotypes is different in all αβ-tubulin heterodimers. The color code for β-tubulin is yellow for tubulin 1SA0, red for isotype βI, light brown for isotype βIIa, violet for isotype βIII, chocolate for isotype βIVa, orange for isotype βIVb, grey for isotype βV and light_green for isotype βVI. Here, indanocine and GTP is shown in stick model and the color code for indanocine and GTP is same as shown in Fig 1.(A) tubulin 1SA0-indanocine complex (B) αβI tubulin isotype-indanocine complex. (C) αβIIa tubulin isotype-indanocine complex (D) αβIII tubulin isotype-indanocine complex (E) αβIVa tubulin isotype-indanocine complex (F) αβIVb tubulin isotype-indanocine complex (G) αβV tubulin isotype-indanocine complex. (H) αβVI tubulin isotype-indanocine complex. Indanocine prefers the αβ-tubulin interface binding pocket in all human αβ-tubulin isotypes.

The binding mode and interactions of indanocine with tubulin 1SA0 and seven different human αβ-tubulin isotypes were examined by molecular docking studies (Fig 3A–3H). In different αβ-tubulin isotypes, indanocine prefers to bind at the αβ-tubulin interface binding pocket i.e. colchicine binding site (Fig 3A–3H). In all the αβ-tubulin isotypes-indanocine complexes, the dimethylphenol group is immersed inside the binding pocket of β-tubulin, while the dimethoxyaniline group of indanocine is located at the interface cavity of αβ-tubulin (Fig 3A–3H). This dimethoxyaniline group of indanocine forms hydrogen bonding interactions with both the residues of α-tubulin and β-tubulin (Table 1). The lowest binding energy docked conformations of indanocine are shown in (Fig 3A–3H). Indanocine shows differences in binding conformations and energy with respect to the residue composition variations in and around the binding pocket of different αβ-tubulin isotypes (Fig 3A–3H and Table 1). The binding energy of indanocine with tubulin 1SA0 and different αβI, αβIIa, αβIII, αβIVa, αβIVb, αβV, and αβVI tubulin isotypes are -8.09, -9.09, -8.07, -8.30, -7.81, -8.73, -8.10 and -8.85 kcal/mol respectively (Table 1).

**Table 1:** Binding energy as well as interactions of indanocine with tubulin 1SA0 and different human αβ tubulin isotypes after molecular docking.

Tubulin Isotypes	Binding energy (kcal/mol)	Hydrogen bonding interactions			Figure references
		Atoms involved	Distance (Å)	Angle (Degree)	
tubulin	-8.09	Ala-315NH…O-Ind	2.18	132.08	Fig 3A, S9A Fig
1SA0		Lys-350CH…O-Ind	2.10	121.40	
		Thr-179-O…HN-Ind	2.16	148.31	
		Asn-101-NH…O-Ind	2.63	145.84	
αβI	-9.09	Ile-236-O…HO-Ind	2.22	103.54	Fig 3B, S9B Fig
		Leu-246-O…HN-Ind	2.00	130.95	
		Lys-252-H…N-Ind	2.54	151.14	
		Ind-O…H-Leu-246	2.14	152.37	
		Asn101-NH…O-Ind	2.92	132.15	
αβIIa	-8.07	Lys-252-NH…O-Ind	2.42	158.46	Fig 3C, S9C Fig
		Lys-350-2BH…O-Ind	1.90	173.19	
		Thr-179-O…HN-Ind	1.98	125.69	
		Asn-101-NH…O-Ind	2.88	141.62	
αβIII	-8.30	Leu-246-O…HN-Ind	2.10	98.13	Fig 3D, S9D Fig
		Lys-252-N…HN-Ind	2.61	127.94	
		Tyr-169-O.....HO-Ind	2.00	176.83	
αβIVa	-7.81	Cys-239-SH…O-Ind	1.84	170.83	Fig 3E, S9E Fig
		Lys-252-NH…O-Ind	2.07	167.76	
		Ser-178-O…HN_Ind	2.26	129.35	
		Ser-178-O…HN-Ind	2.20	110.70	
αβIVb	-8.73	Leu-240NH…O-Ind	2.38	126.85	Fig 3F, S9F Fig
		Val-236-O…HO-Ind	1.73	167.99	
		Cys-239-NH…O-Ind	2.90	98.70	
		Leu-246-CH…O-Ind	2.23	129.32	
		Ala-248-NH…O-Ind	2.70	107.0	
		Asn-256-HN…O-Ind	2.58	98.06	
		Ser-178-OH…O-Ind	2.19	161.91	
αβV	-8.10	Ala-315-O…HO-Ind	1.88	126.21	Fig 3G, S9G Fig
		Lys-252-CH…O-Ind	2.29	139.33	
		Asn-101-NH…O-Ind	2.17	152.54	
		Thr-179-O…HN-Ind	2.20	101.10	
		Val-180-N…HN-Ind	2.80	123.10	
αβVI	-8.85	Tyr-169-O…HO-Ind	2.06	164.22	Fig 3H, S9H Fig
		Lys-252-NH…N-Ind	1.97	123.25	
		Lys-350-CH…O-Ind	2.72	164.94	
		Asn256-N….HN-Ind	2.60	134.00	

The analysis of hydrogen bonding interactions of αβ-tubulin isotypes-indanocine docked complexes shows differences in the hydrogen bonding interactions within the interface binding pocket of different αβ-tubulin isotypes (Table 1). The analysis of tubulin 1SA0-indanocine complex (Fig 3A) shows that the indanocine forms hydrogen bonding interactions with residues Ala-315 (2.18Å), Lys-350 (2.10Å) of β-tubulin, and Thr-179 (2.16Å) and Asn-101 (2.63Å) of α-tubulin (S9A Fig and Table 1). Here, Ala-315, interact with the dimethylphenol group of indanocine and Lys-350, Thr-179 and Asn-101 interact with the dimethoxyaniline group of indanocine (S9A Fig). The analysis of αβI-tubulin isotype-indanocine complex (Fig 3B) shows that indanocine forms hydrogen bonding interactions with the residues Ile-236(2.22Å), Leu-246(2.00Å), Leu-246(2.14Å), Lys-252 (2.54Å) of β-tubulin, and Asn-101(2.92Å) of T5-loop of α-tubulin (S9B Fig and Table 1). Here, Ile-236 interacts with the dimethylphenol group and Leu-246, Lys-252 of β-tubulin and Asn-101 of α-tubulin interacts with the dimethoxyaniline group of indanocine. Next, the analysis of αβIIa-tubulin isotype-indanocine complex (Fig 3C) shows that indanocine forms hydrogen bonding interactions with Lys-252(2.42Å), and Lys-350(1.90Å) of β-tubulin, and with Thr-179(1.98Å) and Asn-101(2.88Å) of T5-loop of α-tubulin (S9C Fig). Here, Lys-350 interacts with the dimethylphenol group of indanocine and Lys-252 of β-tubulin, and Thr-179 and Asn-101 of α-tubulin interacts with the dimethoxyaniline group of indanocine (S9C Fig).

Further analysis of molecular docking complex of αβIII-tubulin isotype-indanocine (Fig 3D) shows that indanocine makes bonding interactions with residues Leu-246 (2.10Å), Lys-252(2.61Å) and Tyr-169(2.00Å) of β-tubulin (S9D Fig and Table 1). Here, Leu-246 and Lys-252 interact with the dimethoxyaniline group of indanocine and Tyr-169 interacts with a dimethylphenol group of indanocine (S9D Fig). Next, the analysis of αβIVa-tubulin isotype-indanocine complex (Fig 3E) shows that indanocine forms hydrogen bonding interactions with residues Cys-239 (1.84Å), Lys-252 (2.07Å) of β-tubulin and Ser-178 (2.26Å) and Ser-178(2.20Å) of T5-loop of α-tubulin (S9E Fig and Table 1). Here, Cys-239 interacts with the dimethylphenol group, and Lys-252 and Ser-178 interact with the dimethoxyaniline group of indanocine (S9E Fig and Table 1). The analysis of αβIVb-tubulin isotype and indanocine (Fig 3F) complex shows that indanocine forms hydrogen bonding interactions with residues Val-236 (1.73Å), Cys-239 (2.90Å), Leu-240 (2.38Å), Leu-246 (2.23Å), Ala-248(2.70Å) and Asn-256 (2.58Å) of β-tubulin and Ser-178 (2.19Å) of T5-loop of α-tubulin (S9F Fig and Table 1). The amino acids Val-236, Cys-239, Leu-240 interact with the dimethylphenol group and Leu-246, Ala-248, Asn-256 and Ser-178 interact with the dimethoxyaniline group of indanocine (S9F Fig and Table 1). Afterwards, an analysis of the docking complex of αβV-tubulin isotype-indanocine (Fig 3G) shows that indanocine forms hydrogen bonding interactions with residues Ala-315 (1.88Å), Lys-252 (2.29Å) of β-tubulin, Asn-101 (2.17Å), and Val-180 (2.80Å) and Thr-179 (2.20Å) of α-tubulin. Here, Ala-315 interacts with the dimethylphenol group of indanocine which is immersed inside the binding pocket of β-tubulin, and Lys-252, Asn-101, Val-180, Thr-179 interact with the dimethoxyaniline group of indanocine (S9G Fig and Table 1). Finally, analysis of αβVI-tubulin isotype and indanocine (Fig 3H) complex shows that indanocine forms interactions with residues Tyr-169 (2.06Å), Asn-256 (2.60Å), Lys350 (2.72 Å) and Lys-252 (1.97Å) of β-tubulin (S9H Fig and Table 1). In this complex, indanocine does not form any hydrogen bonding interactions with α-tubulin. Here, Tyr-169 interacts with the dimethylphenol group and Lys-252, Asn-256 and Lys-350 with the dimethoxyaniline group of indanocine.

Molecular docking results suggest that the residue composition variation in and around the indanocine binding pocket results in differences in binding energy, conformation, and hydrogen bonding interactions among the different αβ-tubulin isotypes-indanocine complexes (Fig 3A–3H and Table 1). Thus, our docking studies suggest that Lys-252, Lys-350, Cys-239, Val-236, Ala-248, Leu-246 of β-tubulin and Ala-101, Ser-178, Thr-179 and Val-180 play a key role in the stabilization of indanocine at the interface of all αβ-tubulin heterodimer (S9A–S9H Fig).

Further, we calculated the electrostatic contact potential over the tubulin 1SA0 and seven different β-tubulin isotypes-indanocine complex using PyMol [39] (S10A–S10H Fig). The electrostatic contact potentials show that the dimethylphenol group is immersed inside the cavity of β-tubulin while the dimethoxyaniline group of indanocine is located out of β-tubulin protein. In addition, the effect of residue composition variation in and around the indanocine binding pocket in different β-tubulin isotypes was further elucidated using molecular dynamics simulations and binding free energy calculations.

### Molecular dynamics simulation of αβ tubulin isotypes-indanocine complexes

**Fig 4:**
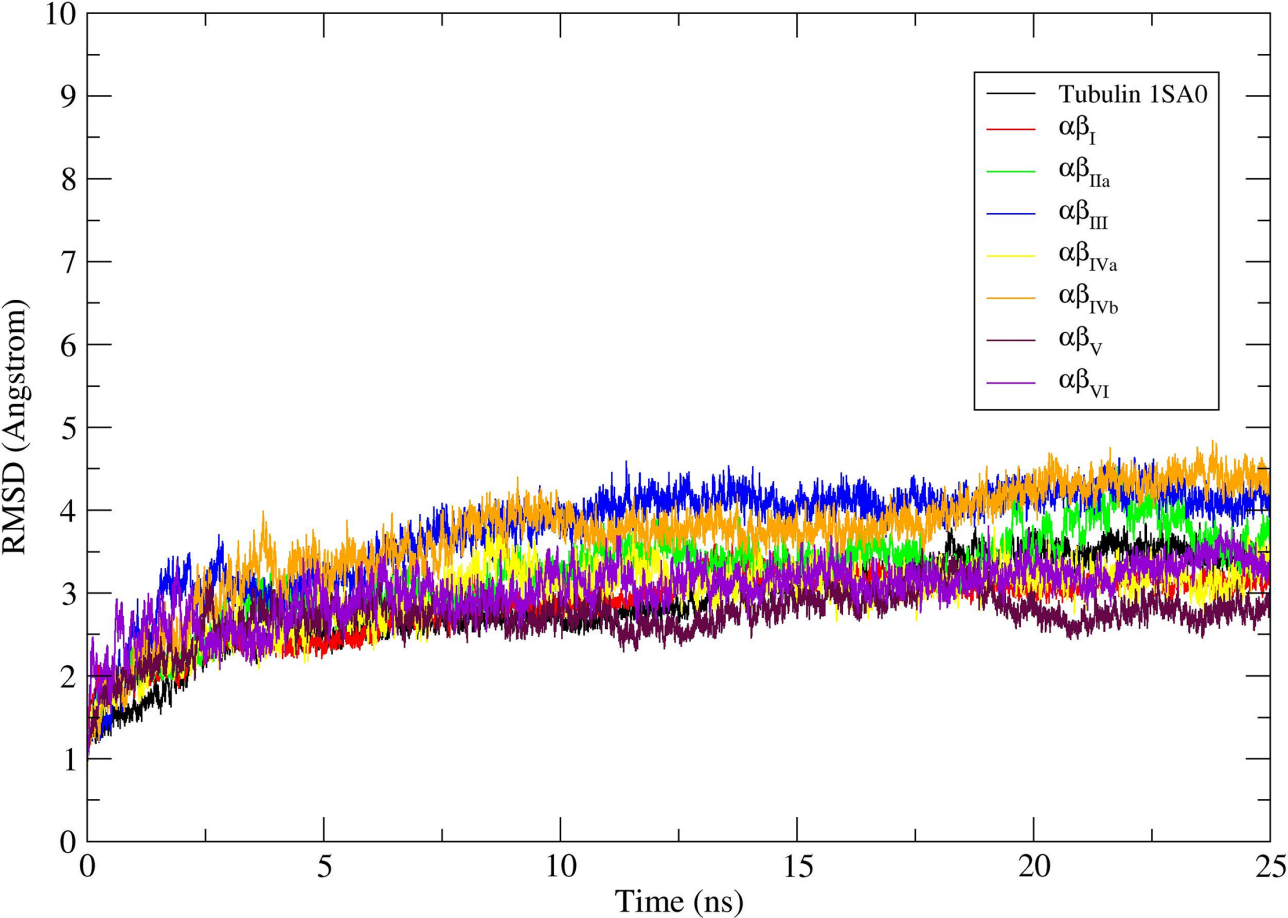
Root mean square deviations (RMSD) corresponding to tubulin 1SA0 and αβ-tubulin isotypes. RMSD correspond to tubulin 1SA0 (black color), αβI(red color), αβIIa (green color), αβIII(blue color), αβIVa (yellow color), αβIVb (orange color), αβV(maroon color), and αβVI (violet color) tubulin heterodimer for 25ns molecular dynamics simulations.

**Fig 5:**
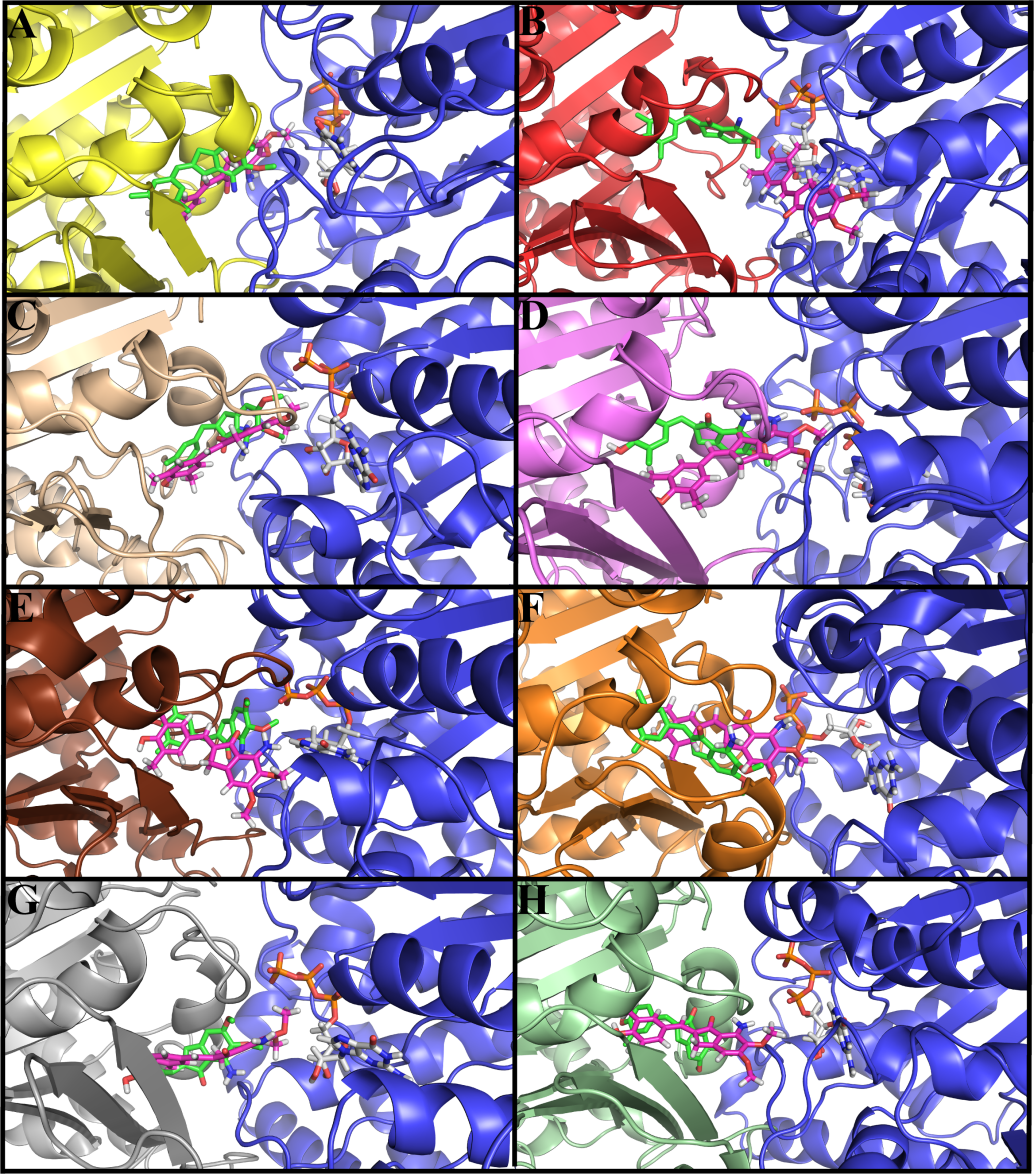
Molecular dynamics simulated structures of αβ tubulin isotypes-indanocine complex. The position of indanocine before and after the simulation is shown for comparison. The color scheme for αβ-tubulin is same as shown in Fig 3. The color scheme for initial docked conformation of indanocine (before MD simulation) shown in green color while indanocine after MD simulation is shown in magenta color. (A) Tubulin1SA0-indanocine complex (B) αβI tubulin isotype-indanocine complex. (C) αβIIa tubulin isotype-indanocine complex (D) αβIII tubulin isotype-indanocine complex (E) αβIVa tubulin isotype-indanocine complex (F) αβIVb tubulin isotype-indanocine complex (G) αβV tubulin isotype-indanocine complex. (H) αβVI tubulin isotype-indanocine complex.

To understand the refined binding mode of αβ-tubulin isotypes with indanocine, we performed molecular dynamics simulations over the lowest energy αβ-tubulin-indanocine docked complexes (Fig 3A–3H) as our starting structure using AMBER12 [28]. The primary analysis was done by looking at the molecular dynamics simulation stability (Fig 4) and analysis of molecular dynamics simulated average structures (Fig 5A–5H).

The Root mean square deviations (RMSD) of Cα backbone atoms of a production molecular dynamics simulations was calculated to examine the stability of the molecular dynamics simulation. The RMSD analysis of all the different αβ-tubulin isotypes-indanocine complexes suggests that all αβ-tubulin-indanocine complexes reached their equilibrium conformation after a time period of 20ns and then retained their stability with fluctuations between 2.5–4.5Å (Fig 4). Molecular dynamics simulation results clearly show that indanocine prefers to bind at the αβ-tubulin interface binding pocket in tubulin 1SA0 and αβIIa, αβIII, αβIVb, αβV, and αβVI tubulin isotypes (S1 Movie, S3 and S4 Movies, S6–S8 Movies) respectively, while in case of tubulin isotype αβI (S2 Movie) and αβIVa (S5 Movie), indanocine is expelled from the αβ-tubulin interface binding cavity. In αβI and αβIVa tubulin isotypes, the T7 loop of β-tubulin moves backward, while the B9 sheet of β-tubulin and T5 loop of α-tubulin also undergo conformational changes which makes ample space at the interface leading to the expulsion of indanocine from the interface of αβ-tubulin isotypes heterodimer (S2 Movie and S5 Movie). Such conformational changes are seen in cases of αβI and αβIVa tubulin isotypes but are not seen on other tubulin isotypes (S1 Movie, S3 and S4 Movies, S6–S8 Movies). The residues present in the B9 sheet, H7 helix, T7 loop and H8 helix of β-tubulin and T5 loop of α-tubulin have important contributions in the binding of indanocine at the interface in other αβ-tubulin isotypes (S1 Movie, S3 and S4 Movies, S6–S8 Movies). A detailed analysis of residues involved in the hydrogen bonding interactions with indanocine is discussed in the next section.

### Analysis of average structure of αβ-tubulin isotypes-indanocine complex

To understand the refined binding mode and interactions of tubulin 1SA0 and different αβ-tubulin isotypes with indanocine, molecular dynamics simulated average structures were analyzed (Fig 5A–5H and Table 2).The RMSD differences of indanocine between the MD simulated ‘starting structure’ (i.e. docked structure) and ‘end structure’ were determined in tubulin 1SA0 and αβI, αβIIa, αβIII, αβIVa, αβIVb, αβV and αβVI tubulin isotypes.,These RMSD differences were found to be 4.11Å, 13.26Å, 2.92Å, 8.98Å, 5.92Å, 6.16Å, 4.63Å and 8.38 Å for tubulin 1SA0 and αβI, αβIIa, αβIII, αβIVa, αβIVb, αβV and αβVI tubulin isotypes respectively. The RMSD analysis shows that indanocine largely deviates from the initial position in the αβI tubulin isotype (Fig 5B) as compared to the other αβ-tubulin-indanocine complexes. The detailed hydrogen bonding interactions of αβ-tubulin isotypes with indanocine are listed in Table 2.

**Table 2:** RMSD and hydrogen bonding interactions of different αβ-tubulin isotypes with indanocine after molecular dynamics simulation.

Tubulin Isotypes	RMSD after MD	Hydrogen bonding interactions			Figure reference
		Atoms involved	Distance (Å)	Angle (Degree)	
tubulin	4.11	Cys-239-S…HC-Ind	3.00	163.80	Fig 5A, S11A Fig
1SA0		Lys-350-CH….O-Ind	2.28	118.39	
		Asn-256-CH…O-Ind	2.82	115.36	
		Lys-252-CH….O-Ind	2.77	152.67	
		Asn-101-O…HC-Ind	2.91	163.50	
		Thr-179-O. . . .HN-Ind	1.86	162.50	
αβI	13.26	Pro-222-O…HN-Ind	1.88	172.56	Fig 5B, S11B Fig
		Val-177-CH…O-Ind	2.90	161.7	
		GTP-O_3_P…HO-Ind	1.81	142.74	
αβIIa	2.92	Asn256-NH…O-Ind	2.80	157.53	Fig 5C, S11C Fig
		Asn256-NH…N-Ind	2.96	142.01	
		Lys252-CH…N-Ind	2.68	143.25	
		Leu246-O.....HC-Ind	3.03	146.82	
		Ala-180-HC….N-Ind	2.99	147.80	
		Asn-101-CH…O-Ind	1.81	163.62	
αβIII	8.98	Val-349-O….HC-Ind	2.71	110.00	Fig 5D, S11D Fig
		Asn-247-O….HC-Ind	2.50	114.10	
		Asn-247-NH…O-Ind	2.36	142.57	
		Asp-249-NH. . . .O-Ind	1.90	172.51	
		Ind-NH….O_3_P-GTP	2.07	154.18	
αβIVa	5.92	Lys-252-CH….O-Ind	2.36	146.74	Fig 5E, S11E Fig
		Lys-252-NH…O-Ind	2.90	121.73	
		Val-180-CH…O-Ind	2.60	129.0	
		Ind-NH…N-GTP	2.13	163.38	
αβIVb	6.16	Cys-239-SH….O-Ind	2.39	158.87	Fig 5F, S11F Fig
		Lys-252-NH…O-Ind	1.93	149.29	
		Thr-351-O….HC-Ind	2.71	114.70	
		Ind-NH…O_1_P-GTP	1.85	166.31	
		Lys-350-NH..O_2_P-GTP	1.73	156.19	
αβV	4.63	Ala-315-O…HC-Ind	2.90	133.40	Fig 5G, S11G Fig
		Thr-351-O…HC-Ind	2.91	124.40	
		Leu-246-CH…O-Ind	2.49	130.22	
		Asn-101-NH…O-Ind	2.35	134.81	
		Ind-O…HC6-GTP	2.70	115.20	
αβVI	8.38	Val-236-O…HO-Ind	2.06	129.91	Fig 5H, S11H Fig
		Thr-315-O…HC-Ind	3.00	142.70	
		Asp-249-NH…O-Ind	2.59	159.19	
		Ser-178-NH—O-Ind	2.10	156.21	
		Ser-178-O…HN-Ind	2.17	153.87	
		Arg221-NH.....O-Ind	2.15	135.47	

The analysis of tubulin 1SA0-indanocine complex (Fig 5A) shows that indanocine makes hydrogen bonding interactions with residue Cys-239(3.00Å), Lys-350(2.28Å), Lys-252 (2.77Å), and Asn-256 (2.82Å) of β-tubulin and Asn-101(2.91Å) and Thr-179(1.86Å) of α-tubulin Table 2. Here, Lys-350, Asn-256, Lys-252, Asn-101, and Thr-179 interact with the dimethoxyaniline group of indanocine and only Cys-239 interacts with the dimethylphenol group of indanocine (S11A Fig). Analysis of the average structure of αβI tubulin isotype-indanocine complex (Fig 5B) shows that the hydrogen bonding interaction of indanocine occurs with only α-tubulin residue Pro-222(1.88Å), Val-177 (2.90Å) as well as with O_3_P of GTP (1.81Å) (Table 2). In the αβI tubulin, indanocine is expelled from the binding pocket and moves towards the surface of αβ-tubulin interface (S1 Movie). Here, the dimethoxyaniline group of indanocine interacts with Pro-222 and Val-177 of α-tubulin whereas the dimethylphenol group interacts with the O_3_P atom of GTP (S11B Fig and Table 2).

The MD simulated αβIIa-indanocine complex (Fig 5C) shows the hydrogen bonding interaction (Table 2) of indanocine with residues Asn-256 (2.80Å), Asn-256(2.96Å) and Leu-246(3.03Å) of β-tubulin, and Lys-252(2.68Å), Asn-101(1.81Å) and Ala-180(2.99Å) of α-tubulin (S11C Fig). Here, the residues Leu-246, Lys-252, Asn-256, Asn-101 and Ala-180 interact with dimethoxyaniline group of indanocine (S11C Fig). Further, in the αβIII-indanocine complex (Fig 5D), indanocine makes interactions with Val-349(2.71Å), Asn-247 (2.50Å), Asn-247(2.36Å), and Asp-249(1.90Å) as well as with O_3_P of GTP (2.07Å) (Table 2). Here, Val-349 interacts with the dimethylphenol group and O_3_P of GTP, Asn-247 and Asp-248 interact with the dimethoxyaniline group of indanocine (S11D Fig). Analysis of the MD simulated average structure of αβIVa-indanocine (Fig 5E) complex shows that indanocine makes interactions with Lys-252(2.36Å), Lys-252(2.90Å) of β-tubulin and Val-180(2.60Å) and adenosine of GTP (2.13Å) (Table 2). Lys-252, Val-180, and GTP interact with the dimethoxyaniline group of indanocine (S11E Fig). In the αβIVa-indanocine complex (Fig 5E), indanocine is expelled from the αβ-tubulin interface, as the T7-loop moves backward and B9 sheet and a T5 loop of α-tubulin also undergoes conformational changes which make enough space for indanocine to get expelled from the interface cavity (S5 Movie).

In the αβIVb-indanocine MD simulated complex (Fig 5F), indanocine shows hydrogen bonding interactions with residues Cys-239(2.39Å), Lys-252(1.93Å), Lys-350(1.73Å), and Thr-351 (2.71Å) and O_1_P of GTP (1.85Å) (Table 2). Cys-239 interacts with the dimethylphenol group of indanocine whereas Lys-252, Lys-350, and Thr-351 of β-tubulin and O_2_P of GTP interact with the dimethoxyaniline group of indanocine (S11F Fig). Next, the analysis of αβV-indanocine MD simulated complex (Fig 5G) shows that, indanocine forms hydrogen bonding interactions with the residues Ala-315(2.90Å), Thr-351(2.91Å), Leu-246 (2.49Å) of β-tubulin, and Asn-101(2.35Å) of α-tubulin as well as with the HC6 of GTP(2.70Å) (Table 2). Here, Ala-315 interact with the dimethylphenol group of indanocine and Thr-351, Leu-246, Asn-101, and GTP interact with the dimethoxyaniline group of indanocine (S11G Fig). Finally, the analysis of αβVI-indanocine (Fig 5H) complex shows hydrogen bonding interactions between indanocine with residues Val-236(2.06Å), Thr-315(3.00Å), Asp-249(2.59Å) of β-tubulin, and Ser-178(2.10Å), Ser-178(2.17Å) and Arg-221(2.15Å) of α-tubulin (Table 2). Here, Val-236 and Thr-315 interact with the dimethylphenol group of indanocine and Ser-178, and Arg-221 and Asp-249 interact with the dimethoxyaniline group of indanocine (S11H Fig).

The analysis of molecular dynamics simulated average structures of different αβ-tubulin-indanocine complexes shows that the dimethoxyaniline group of indanocine interacts with α-tubulin and β-tubulin residues, while the dimethylphenol group of indanocine interacts with β-tubulin residues except in case of only αβI-indanocine complex. In αβI-indanocine complex, indanocine is expelled from its initial binding pose and moves towards the surface of α-tubulin (S2 Movie). Overall, the residues at the indanocine binding pocket such as Cys-239, Leu-246, Lys-252, Ala-315, Lys-350 and Thr-351 of β-tubulin, and Asn-101, Ser-178, Thr-179, Val-180 of T5-loop of α-tubulin play an important role in the binding of indanocine at the interface of αβ-tubulin isotypes. However, the αβI-indanocine complex does not show any such bonding interactions, as indanocine moves from its initial binding position (Table 2 and S2 Movie). In tubulin 1SA0 and human tubulin isotypes αβIIa, αβIII, αβIVb, αβV, and αβVI, the T7 loop of β-tubulin move forward and the B9-sheet of β-tubulin and T5 loop of α-tubulin move backward, which makes enough space to adopt indanocine at the αβ-interface cavity (S1 Movie, S3 and S4 Movies, S6–S8 Movies). Further, we calculated the electrostatic potentials to show the binding mode of indanocine after molecular dynamics simulation (S12A–S12H Fig). The electrostatic potential surface shows that indanocine is located inside the binding cavity of human β-tubulin isotypes except in βI-tubulin (S12B Fig). Similar to hydrogen bonding interactions, the electrostatic and van der Waals interactions also play a role in the protein-ligand complex stabilization. Therefore, the MM-GBSA binding free energy calculations were used to further analyze the binding free energy difference between different αβ-tubulin isotype-indanocine complexes.

### Binding energy calculations

As reported earlier [10], the binding free energies for different αβ-tubulin isotypes with indanocine were calculated ignoring the entropic contribution to the binding free energy (Table 3). The estimated binding free energies (ΔE_bind_) of tubulin 1SA0 and different αβI, αβIIa, αβIII, αβIVa, αβIVb, αβV, and αβVI tubulin isotypes with indanocine are -49.90, -41.39, -44.03, -43.47, -41.50, -44.57, -42.97, and -50.70 kcal/mol, respectively (Table 3). The αβVI has the highest binding free energy for indanocine, whereas αβI has the lowest binding free energy among the other αβ-tubulin isotypes. The binding free energy decreases is in the order of αβVI > αβIIb > αβIVb > αβIIa > αβIII > αβV > αβIVa > αβI. The lower binding free energy of indanocine for αβI-tubulin isotype is due to maximum residue changes in the binding pocket of βI-tubulin such as Val236-Ile, Cys239-Ser, Ala315-Cys, Val316-Ile, and Thr351-Val as compared to other tubulin isotypes (Fig 2). However, indanocine exhibits a good binding affinity for αβVI, αβIIb, αβIVb, αβIIa, αβIII, and αβV (Table 3). The van der Waal (ΔE_vdw_) and electrostatic (ΔE_ele_) interactions are important for the binding of the protein-ligand complex. Here, van der Waal interactions make the highest contribution towards the binding free energy (Table 3), while the solvation energy (E_sol_) is unfavorable for binding of ligand. The αβI- tubulin isotype shows the lowest van der Waals interaction energy in comparison to the other αβ-tubulin isotype-indanocine complexes. The net binding free energy is decided by a competition between *E*_*gas*_ and *E*_*sol*_, and is the lowest for αβI-tubulin isotype (Table 3).

**Table 3:** Binding energy of different αβ-tubulin isotypes with indanocine.

Tubulin isotypes	Δ*E*_*vdw*_	Δ*E*_*ele*_	Δ*E*_*gas*_	Δ*E*_*sol*_	^*a*^Δ*E*_*bind*_
tubulin 1SA0	-56.85	-19.20	-76.05	26.15	-49.90
αβI	-42.26	-34.22	-76.48	35.09	-41.39
αβIIa	-46.27	-24.74	-71.01	26.98	-44.03
αβIII	-47.10	-19.74	-66.84	23.37	-43.47
αβIVa	-46.22	-10.93	-57.15	15.65	-41.50
αβIVb	-47.66	-23.58	-71.24	26.67	-44.57
αβV	-51.06	-4.81	-55.87	12.90	-42.97
αβVI	-52.21	-20.99	-73.20	22.50	-50.70

^*a*^Δ*E*_*bind*_ = Δ*E*_*gas*_ + Δ*E*_*sol*_ = (Δ*E*_*vdw*_ + Δ*E*_*ele*_) + (Δ*E*_*polar*_ + Δ*E*_*nonpolar*_)

## Conclusion

In this study, the binding affinity of indanocine with tubulin 1SA0 and seven human tubulin isotypes αβI, αβIIa, αβIII, αβIVa, αβIVb, αβV, and αβVI was investigated using sequence analysis, homology modeling, molecular docking, molecular dynamics simulations and binding free energy calculations. The residue compositions were found to be different at the indanocine binding pocket of human βI, βIIa, βIII and βVI tubulin isotypes, whereas no such differences were found in the βIVa, βIVb and βV tubulin isotypes.

Further, molecular docking results show that indanocine prefers to bind at the interface of all αβ-tubulin isotypes i.e. at the colchicine binding site, as observed in the previous experimental study [21]. Indanocine shows different binding mode and binding energy for different αβ-tubulin isotypes; this might be due to the residue composition changes in and around the binding pocket of β-tubulin isotypes. The residues in the H7-Helix (Cys-239, Ile-236), T7-loop (Leu-246, Ala-248), H8-helix (Lys-252, Asn-256) and B9-sheet (Lys-350) of β-tubulin and T5-loop (Ser-178, Thr-179, Val-180) of α-tubulin are involved in the hydrogen bonding interactions with indanocine.

Molecular dynamics simulations were performed on αβ-tubulin isotype-indanocine docked complexes, to further investigate the effect of residue composition differences on the binding of indanocine. Our molecular dynamics simulations results show that indanocine is completely adopted inside the binding pocket of tubulin 1SA0 and αβIIa, αβIII, αβIVb, αβV and αβVI tubulin isotypes, whereas it is expelled from the interface of αβI, and αβIVa-tubulin isotype. Here, the T7-loop of β-tubulin moves backward; meanwhile the B9 sheet of β-tubulin and T5-loop of α-tubulin shows conformational change. This leads to making an ample space at the interface of αβI, and αβIVa tubulin isotypes which is helpful to expel indanocine from the interface cavity. Whereas in case of other αβ-tubulin isotypes-indanocine complexes, the T7 loop moves forward which helps to adopt indanocine at the binding pocket. Further, binding free energy calculations show that the tubulin isotypes αβIIa, αβIII, αβIVa, αβIVb, αβV and αβVI have the highest binding free energy and αβI-tubulin isotype has the lowest binding free energy for indanocine. One of the reasons behind the less binding free energy of αβI-tubulin isotype toward indanocine might be due to maximum residue changes at the binding site.

Thus, our present computational study provides a detailed understanding of the molecular interactions of human αβ-tubulin isotypes with indanocine and provides insight for designing superior indanocine analogues with isotype specificity. These superior analogues can be valuable in the treating patients with advanced carcinomas which exhibit tubulin isotype specificity or can be helpful in developing personalized medicines for cancer patients.

## Supporting information

S1 Text Stereo-chemical Quality Analysis of different αβ-tubulin isotypes. (DOC)

S1 Fig PROCHECK plot for tubulin 1SA0. (PDF)

S2 Fig PROCHECK plot for αβI-tubulin isotypes. (PDF)

S3 Fig PROCHECK plot for αβIIa-tubulin isotypes. (PDF)

S4 Fig PROCHECK plot for αβIII-tubulin isotypes. (PDF)

S5 Fig PROCHECK plot for αβIVa-tubulin isotypes. (PDF)

S6 Fig PROCHECK plot for αβIVb-tubulin isotypes. (PDF)

S7 Fig PROCHECK plot for αβV-tubulin isotypes. (PDF)

S8 Fig PROCHECK plot for αβVI-tubulin isotypes. (PDF)

S9 Fig Hydrogen bonding interactions of indanocine with different αβ-tubulin isotypes after molecular docking. **(A)** Tubulin 1SA0 and indanocine complex, indanocine interacts with Ala-315 (2.18Å), Lys-350 (2.10Å) of β-tubulin, and Thr-179 (2.16Å) and Asn-101 (2.63Å) of α-tubulin (B) αβI-tubulin and indanocine complex, indanocine interacts with Ile-236(2.22Å), Leu-246(2.00Å), Leu-246(2.14Å), Lys-252 (2.54Å) of β-tubulin, and Asn-101(2.92Å) of α-tubulin **(C)** αβIIa tubulin isotype and indanocine complex, here indanocine interacts with Lys-252(2.42Å), and Lys-350(1.90Å) of β-tubulin, and with Thr-179(1.98Å) and Asn-101(2.88Å) of T5-loop of α-tubulin **(D)** αβ_III_ tubulin isotype and indanocine complex, indanocine interacts with Leu-246 (2.10Å), Lys-252(2.61Å) and Tyr-169(2.00Å) of β-tubulin **(E)** αβIVa tubulin isotype and indanocine complex, indanocine interacts with Cys-239 (1.84Å), Lys-252 (2.07Å) of β-tubulin and Ser-178 (2.26Å) and Ser-178(2.20Å) of T5-loop of α-tubulin **(F)** αβIVb tubulin isotype and indanocine complex, indanocine interacts with residue Val-236 (1.73Å), Cys-239 (2.90Å), Leu-240 (2.38Å), Leu-246 (2.23Å), Ala-248(2.70Å) and Asn-256 (2.58Å) of α-tubulin and Ser-178 (2.19Å) of T5-loop of β-tubulin **(G)** αβV tubulin isotype and indanocine complex, indanocine interacts with Ala-315 (1.88Å), Lys-252 (2.29Å) of β-tubulin, Asn-101 (2.17Å), and Val-180 (2.80Å) and Thr-179 (2.20Å) of α-tubulin. and **(H)** αβVI tubulin isotype and indanocine complex, indanocine interacts with Tyr-169 (2.06Å), Asn-256 (2.60Å) Lys-350 (2.72Å) and Lys-252 (1.97Å) of β
-tubulin.
(TIF)

S10 Fig The electrostatic contact potential of different β-tubulin isotypes with docked indanocine. The red, blue and white color represents the negative, positive and neutral electrostatic potentials, respectively. The indanocine bind at the interface of the cavity of β-tubulin in all the tubulin isotypes. indanocine is shown in green color; oxygen, nitrogen, and hydrogen atoms are shown in red, blue, and grey colors respectively. **(A)** Tubulin 1SA0 and indanocine complex **(B)** βI-tubulin and Indanocine complex **(C)** βIIa tubulin isotype and indanocine complex, **(D)** βIII tubulin isotype and indanocine complex **(E)** βIVa tubulin isotype and indanocine complex **(F)** βIVb tubulin isotype and indanocine complex **(G)** βV tubulin isotype and indanocine complex and **(H)** βVI tubulin isotype and indanocine complex.
(TIF)

S11 Fig Hydrogen bonding interactions of indanocine with different αβ-tubulin isotypes after molecular dynamics simulation. **(A)** Tubulin 1SA0 and indanocine complex, indanocine shows interaction with Cys-239(3.00Å), Lys-350(2.28Å), Lys-252 (2.77Å), and Asn-256 (2.82Å) of β-tubulin and Asn-101(2.91Å) and Thr-179(1.86Å) of α-tubulin **(B)** αβI-tubulin and indanocine complex, indanocine shows interaction with Pro-222(1.88Å), Val-177 (2.90Å) as well as with O_3_P of GTP(1.81Å) **(C)** αβIIa tubulin isotype and indanocine complex, indanocine shows interaction with Asn-256 (2.80Å), Asn-256(2.96Å) and Leu-246(3.03Å) of β-tubulin, and Lys-252(2.68Å), Asn-101(1.81Å) and Ala-180(2.99Å) of α-tubulin **(D)** αβIII tubulin isotype and indanocine complex, indanocine shows interaction with Val-349(2.71Å), Asn-247 (2.50Å), Asn-247(2.36Å), and Asp-249(1.90Å) as well as with O_3_P of GTP (2.07Å) **(E)** αβIVa tubulin isotype and indanocine complex, indanocine shows interaction with Lys-252(2.36Å), Lys-252(2.90Å) of β-tubulin and Val-180(2.60Å) and adenosine of GTP (2.13Å) **(F)** αβIVb tubulin isotype and indanocine complex, indanocine shows interaction with Cys-239(2.39Å), Lys-252(1.93Å), Lys-350(1.73Å), and Thr-351 (2.71Å) and O_1_P of GTP (1.85Å) **(G)** αβV tubulin isotype and indanocine complex, indanocine shows interaction with Ala-315(2.90Å), Thr-351(2.91Å), Leu-246 (2.49Å) of β-tubulin, and Asn-101(2.35Å) of α-tubulin as well as with the HC6 of GTP(2.70Å) and **(H)** αβVI tubulin isotype and indanocine complex, indanocine shows interaction with Val-236(2.06Å), Thr-315(3.00Å), Asp-249(2.59Å) of β-tubulin, and Ser-178(2.10Å), Ser-178(2.17Å) and Arg-221(2.15Å) of α-tubulin.
(TIF)

S12 Fig The electrostatic contact potential of indanocine with different β-tubulin isotypes after molecular dynamics simulation. Colour scheme is same as shown in S2 Fig. (A) Tubulin 1SA0 and indanocine complex (B) βI-tubulin and Indanocine complex, here indanocine expelled from the binding pocket (C) βIIa tubulin isotype and indanocine complex, (D) βIII tubulin isotype and indanocine complex (E) βIVa tubulin isotype and indanocine complex (F) βIVb tubulin isotype and indanocine complex (G) βV tubulin isotype and indanocine complex and (H) βVI tubulin isotype and indanocine complex.
(TIF)

S1 Movie MD simulation movie of tubulin 1SA0 and indanocine. (MPG)

S2 Movie MD simulation movie of αβI and indanocine. (MPG)

S3 Movie MD simulation movie of αβIIa tubulin isotype and indanocine. (MPG)

S4 Movie MD simulation movie of αβIII tubulin isotype and indanocine. (MPG)

S5 Movie MD simulation movie of αβIVa and indanocine. (MPG)

S6 Movie MD simulation movie of αβIVb tubulin isotype and indanocine. (MPG)

S7 Movie MD simulation movie of αβV tubulin isotype and indanocine. (MPG)

S8 Movie MD simulation movie of αβVI tubulin isotype and indanocine. (MPG)
