# Supplementary material for: Interaction of microtubule depolymerizing agent indanocine with different human αβ tubulin isotypes
Source: PLoS One. 2018 Mar 27;13(3):e0194934. doi: 10.1371/journal.pone.0194934 (PMC5870988; doi:10.1371/journal.pone.0194934)
Supplement: S1 Text — (DOC) [file pone.0194934.s001.doc]

**Supplementary Text**

**Stereo-chemical Quality Analysis of different αβ-tubulin isotypes:**

The stereo-chemical quality of Tubulin-1SA0 and seven different human αβ-tubulin isotypes was evaluated by using the online programs PROCHECK [1] and VERIFY-3D [2]. The PROCHECK checks the stereo-chemical quality of a three dimensional structure of protein. The Ramachandran plot produced by PROCHECK shows the phi-psi torsion angles for all residues in the protein structure, and the PROCHECK results show the percentage of residues that are present in most favored regions, residues in additional allowed regions, residues in generously allowed regions and residues in disallowed regions. These percentages obtained using PROCHECK for different human αβ-tubulin isotypes are given in Table S1 and S1-S8 Figs. Thus, Ramachandran plots for different human αβ-tubulin isotypes show that the quality of the model is good for further molecular modeling study as maximum number of residues are present in the most favoured regions.

**Table S1. Percentage of residues present in the different regions for different human αβ-tubulin isotypes obtained using PROCHECK**

| **αβ-Tubulin isotypes** | **% of Most favored regions** | **% of Additional allowed regions** | **% of Generously allowed regions** | **% of Disallowed regions** | **Figure**  **reference** |
| --- | --- | --- | --- | --- | --- |
| **Tubulin-1SA0** | 87.2 | 10.0 | 1.8 | 1.0 | S1 Fig |
| **αβI** | 86.7 | 9.2 | 2.9 | 1.2 | S2 Fig |
| **αβIIa** | 86.9 | 9.7 | 2.6 | 0.8 | S3 Fig |
| **αβIII** | 86.2 | 10.0 | 2.9 | 0.9 | S4 Fig |
| **αβIVa** | 86.8 | 9.8 | 2.6 | 0.8 | S5 Fig |
| **αβIVb** | 87.3 | 9.3 | 2.7 | 0.8 | S6 Fig |
| **αβV** | 87.0 | 9.4 | 2.8 | 0.8 | S7 Fig |
| **αβVI** | 87.8 | 8.5 | 2.9 | 0.8 | S8 Fig |

We further checked the quality of the homology models using Verify-3D [2]. The VERIFY-3D scores for αβ_I_, αβ_IIa_, αβ_IIb_, αβ_III_, αβ_IVa_, αβ_IVb_, αβ_V_ and αβ_VI_ were 98.27%, 97.93, 96.76%, 97.81%, 98.16%, 97.92%, 97.92%, and 98.04% respectively, which further confirm the good quality of the models.

In addition, we also calculated the RMSD difference between the template structure i.e. 1SA0.pdb (chain A and B) and energy minimized structure of different human αβ-tubulin isotypes. The RMSD between template 1SA0.pdb and αβI, αβIIa, αβIIb, αβIII, αβIVa, αβIVb, αβV, and αβVI, are 0.65Å, 0.48Å, 0.61Å, 0.51Å, 0.46Å, 0.46Å, 0.49Å, and 0.51Å respectively, which also confirms that the quality of homology models were good for further molecular modeling study.

**S1 Text References**

1. Laskowski R a., MacArthur MW, Moss DS, Thornton JM. PROCHECK: a program to check the stereochemical quality of protein structures. Journal of Applied Crystallography. 1993;26: 283–291. doi:10.1107/S0021889892009944

2. Bowie JU, Lüthy R, Eisenberg D. A method to identify protein sequences that fold into a known three-dimensional structure. Science (New York, NY). 1991;253: 164–170. doi:10.1126/science.1853201
