# Supplementary material for: Interaction of microtubule depolymerizing agent indanocine with different human αβ tubulin isotypes
Source: PLoS One. 2018 Mar 27;13(3):e0194934. doi: 10.1371/journal.pone.0194934 (PMC5870988; doi:10.1371/journal.pone.0194934)
Supplement: S6 Fig — (PDF) [file pone.0194934.s007.pdf]

PROCHECK

Ramachandran Plot

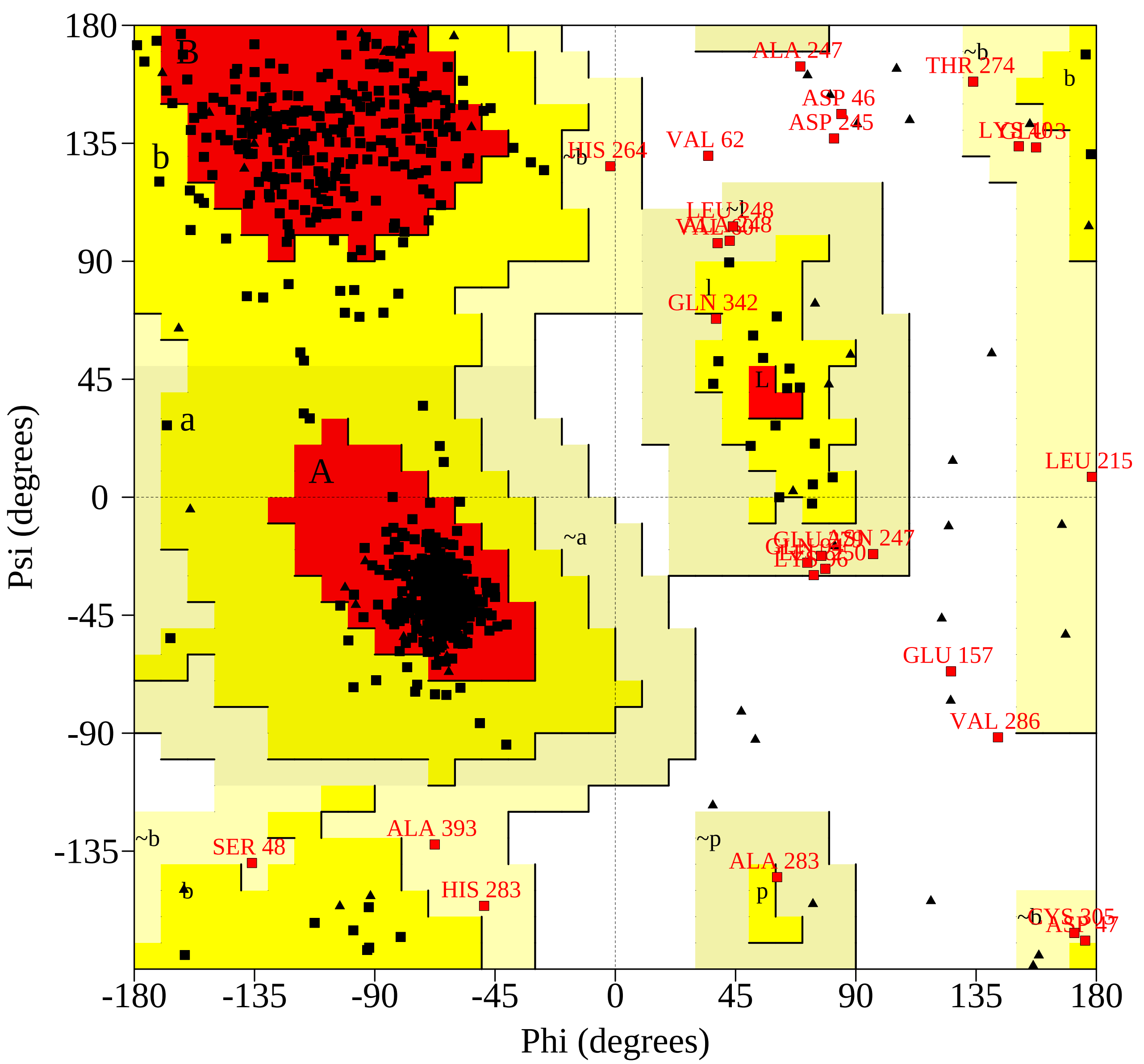

Plot statistics

Residues in most favoured regions [A,B,L]	657	87.3%
Residues in additional allowed regions [a,b,l,p]	70	9.3%
Residues in generously allowed regions [~a,~b,~l,~p]	20	2.7%
Residues in disallowed regions	6	0.8%
	----	------
Number of non-glycine and non-proline residues	753	100.0%
Number of end-residues (excl. Gly and Pro)	8	
Number of glycine residues (shown as triangles)	68	
Number of proline residues	40	

Total number of residues	869	

Based on an analysis of 118 structures of resolution of at least 2.0 Angstroms and R-factor no greater than 20%, a good quality model would be expected to have over 90% in the most favoured regions.
