# Supplementary figures and images for: Interaction of microtubule depolymerizing agent indanocine with different human αβ tubulin isotypes
Source: PLoS One. 2018 Mar 27;13(3):e0194934. doi: 10.1371/journal.pone.0194934 (PMC5870988; doi:10.1371/journal.pone.0194934)

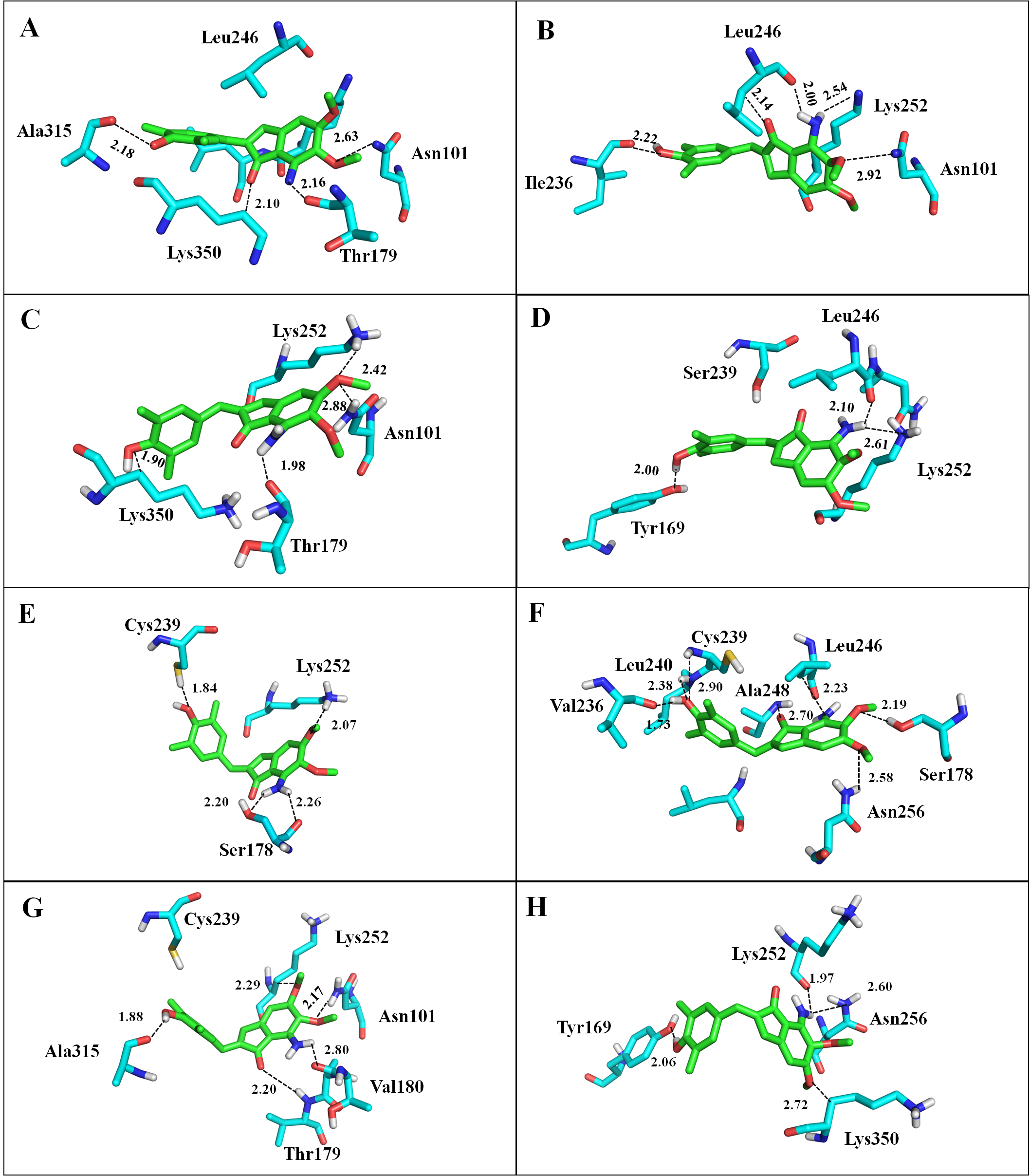

Supplement: S9 Fig — (A) Tubulin 1SA0 and indanocine complex, indanocine interacts with Ala-315 (2.18Å), Lys-350 (2.10Å) of β-tubulin, and Thr-179 (2.16Å) and Asn-101 (2.63Å) of α-tubulin (B) αβI-tubulin and indanocine complex, indanocine interacts with Ile-236(2.22Å), Leu-246(2.00Å), Leu-246(2.14Å), Lys-252 (2.54Å) of β-tubulin, and Asn-101(2.92Å) of α-tubulin (C) αβIIa tubulin isotype and indanocine complex, here indanocine interacts with Lys-252(2.42Å), and Lys-350(1.90Å) of β-tubulin, and with Thr-179(1.98Å) and Asn-101(2.88Å) of T5-loop of α-tubulin (D) αβIII tubulin isotype and indanocine complex, indanocine interacts with Leu-246 (2.10Å), Lys-252(2.61Å) and Tyr-169(2.00Å) of β-tubulin (E) αβIVa tubulin isotype and indanocine complex, indanocine interacts with Cys-239 (1.84Å), Lys-252 (2.07Å) of β-tubulin and Ser-178 (2.26Å) and Ser-178(2.20Å) of T5-loop of α-tubulin (F) αβIVb tubulin isotype and indanocine complex, indanocine interacts with residue Val-236 (1.73Å), Cys-239 (2.90Å), Leu-240 (2.38Å), Leu-246 (2.23Å), Ala-248(2.70Å) and Asn-256 (2.58Å) of α-tubulin and Ser-178 (2.19Å) of T5-loop of β-tubulin (G) αβV tubulin isotype and indanocine complex, indanocine interacts with Ala-315 (1.88Å), Lys-252 (2.29Å) of β-tubulin, Asn-101 (2.17Å), and Val-180 (2.80Å) and Thr-179 (2.20Å) of α-tubulin. and (H) αβVI tubulin isotype and indanocine complex, indanocine interacts with Tyr-169 (2.06Å), Asn-256 (2.60Å) Lys-350 (2.72Å) and Lys-252 (1.97Å) of β -tubulin. (TIF) [file pone.0194934.s010.tif]

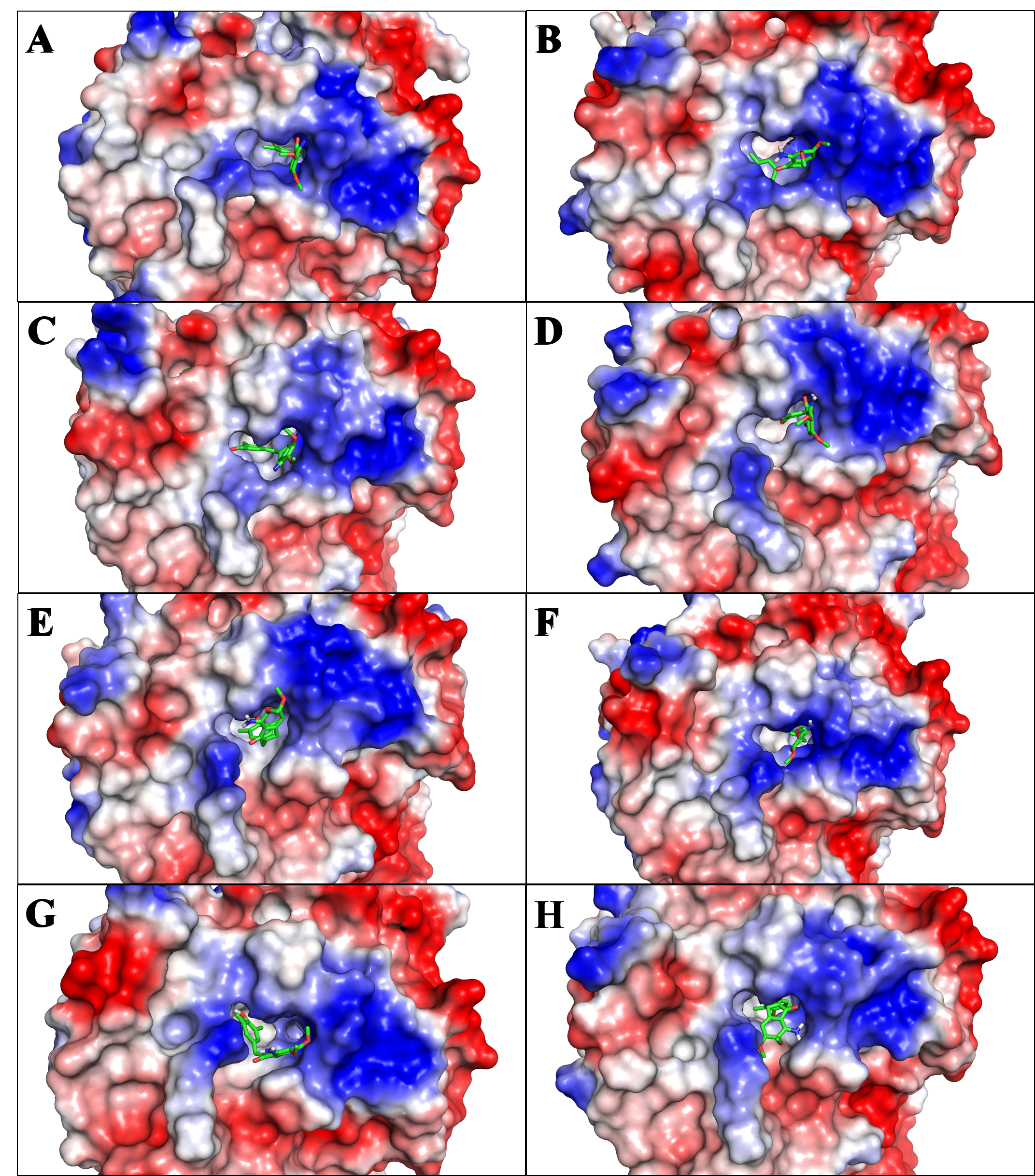

Supplement: S10 Fig — The red, blue and white color represents the negative, positive and neutral electrostatic potentials, respectively. The indanocine bind at the interface of the cavity of β-tubulin in all the tubulin isotypes. indanocine is shown in green color; oxygen, nitrogen, and hydrogen atoms are shown in red, blue, and grey colors respectively. (A) Tubulin 1SA0 and indanocine complex (B) βI-tubulin and Indanocine complex (C) βIIa tubulin isotype and indanocine complex, (D) βIII tubulin isotype and indanocine complex (E) βIVa tubulin isotype and indanocine complex (F) βIVb tubulin isotype and indanocine complex (G) βV tubulin isotype and indanocine complex and (H) βVI tubulin isotype and indanocine complex. (TIF) [file pone.0194934.s011.tif]

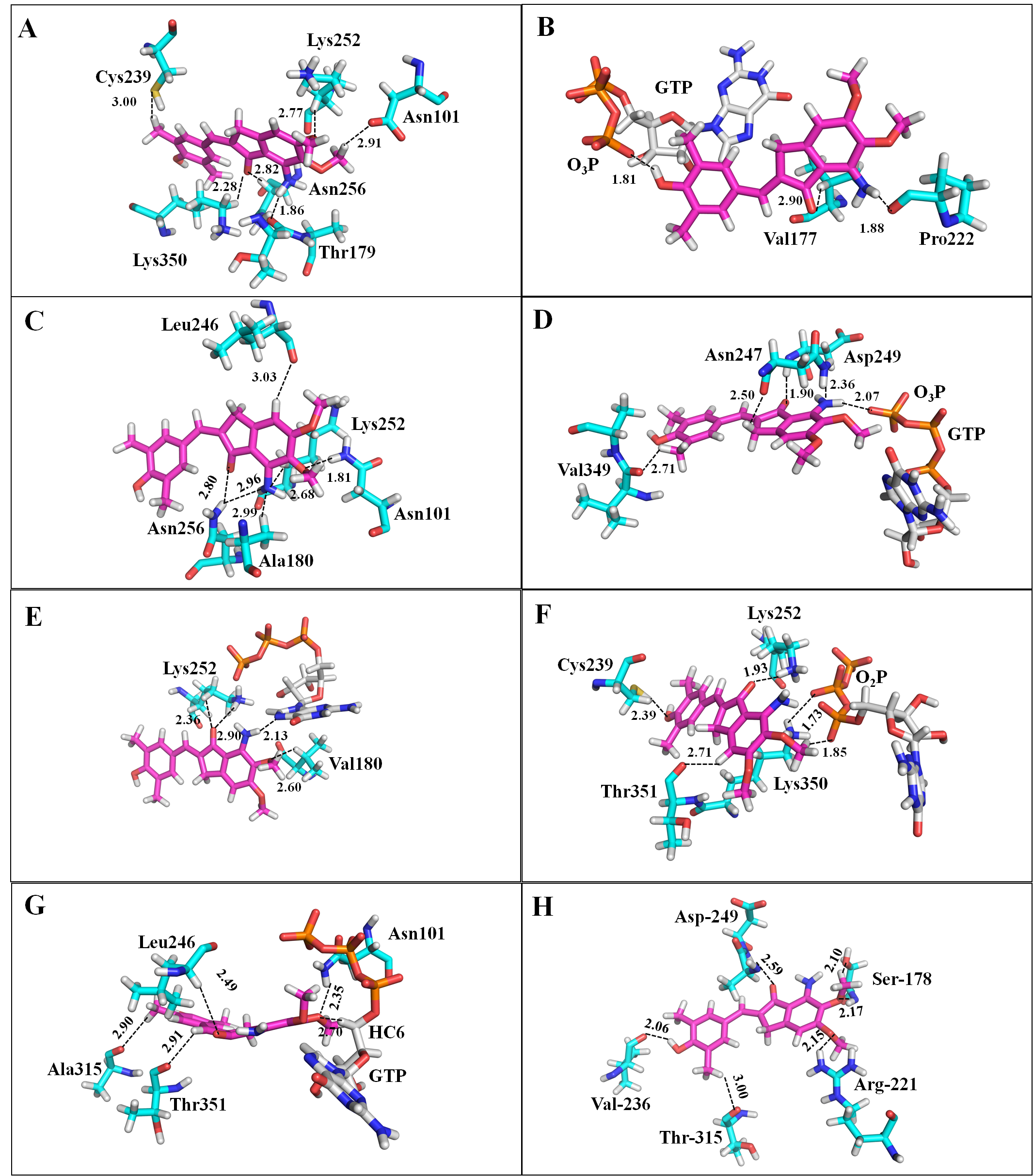

Supplement: S11 Fig — (A) Tubulin 1SA0 and indanocine complex, indanocine shows interaction with Cys-239(3.00Å), Lys-350(2.28Å), Lys-252 (2.77Å), and Asn-256 (2.82Å) of β-tubulin and Asn-101(2.91Å) and Thr-179(1.86Å) of α-tubulin (B) αβI-tubulin and indanocine complex, indanocine shows interaction with Pro-222(1.88Å), Val-177 (2.90Å) as well as with O3P of GTP(1.81Å) (C) αβIIa tubulin isotype and indanocine complex, indanocine shows interaction with Asn-256 (2.80Å), Asn-256(2.96Å) and Leu-246(3.03Å) of β-tubulin, and Lys-252(2.68Å), Asn-101(1.81Å) and Ala-180(2.99Å) of α-tubulin (D) αβIII tubulin isotype and indanocine complex, indanocine shows interaction with Val-349(2.71Å), Asn-247 (2.50Å), Asn-247(2.36Å), and Asp-249(1.90Å) as well as with O3P of GTP (2.07Å) (E) αβIVa tubulin isotype and indanocine complex, indanocine shows interaction with Lys-252(2.36Å), Lys-252(2.90Å) of β-tubulin and Val-180(2.60Å) and adenosine of GTP (2.13Å) (F) αβIVb tubulin isotype and indanocine complex, indanocine shows interaction with Cys-239(2.39Å), Lys-252(1.93Å), Lys-350(1.73Å), and Thr-351 (2.71Å) and O1P of GTP (1.85Å) (G) αβV tubulin isotype and indanocine complex, indanocine shows interaction with Ala-315(2.90Å), Thr-351(2.91Å), Leu-246 (2.49Å) of β-tubulin, and Asn-101(2.35Å) of α-tubulin as well as with the HC6 of GTP(2.70Å) and (H) αβVI tubulin isotype and indanocine complex, indanocine shows interaction with Val-236(2.06Å), Thr-315(3.00Å), Asp-249(2.59Å) of β-tubulin, and Ser-178(2.10Å), Ser-178(2.17Å) and Arg-221(2.15Å) of α-tubulin. (TIF) [file pone.0194934.s012.tif]

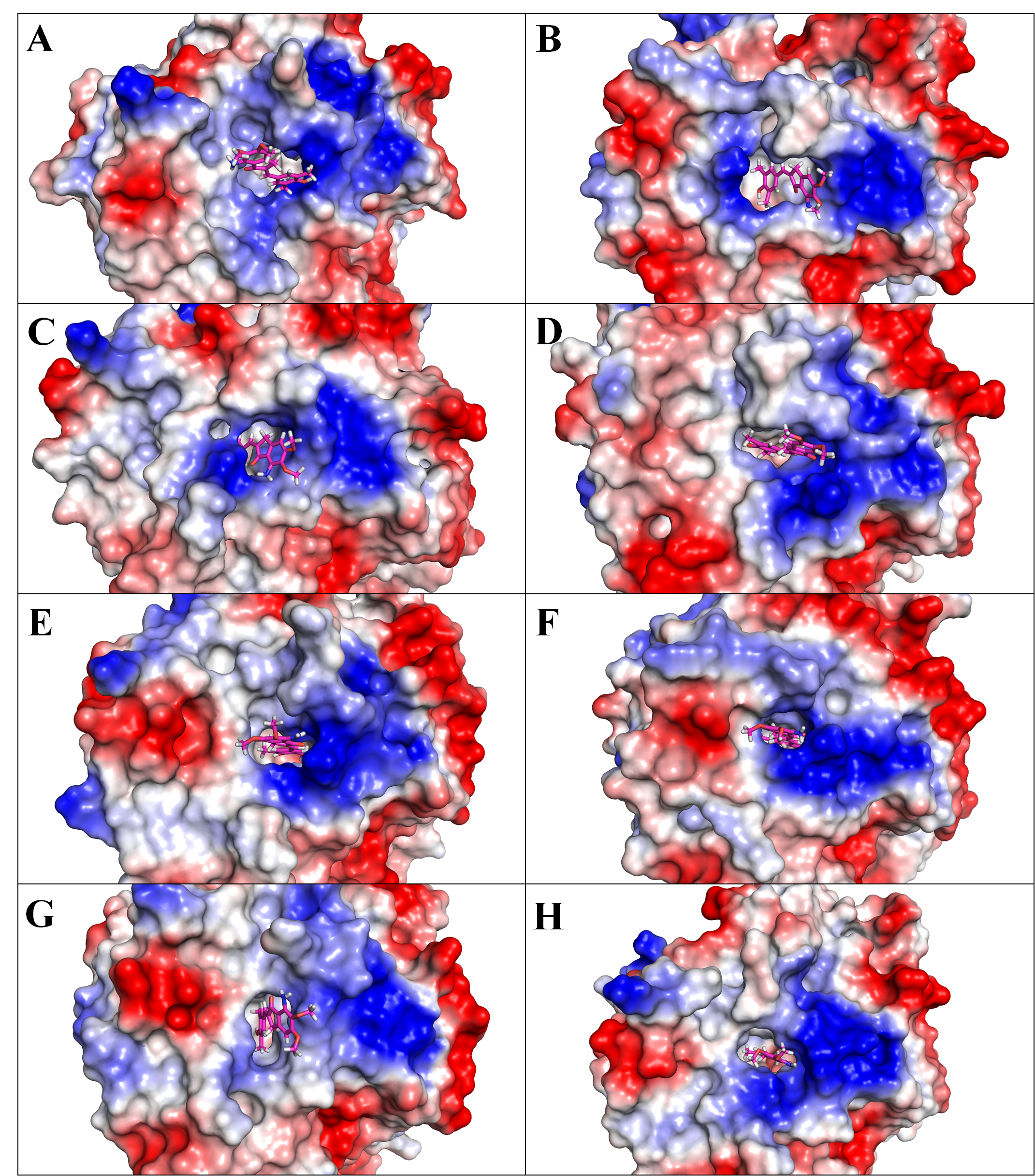

Supplement: S12 Fig — Colour scheme is same as shown in S2 Fig. (A) Tubulin 1SA0 and indanocine complex (B) βI-tubulin and Indanocine complex, here indanocine expelled from the binding pocket (C) βIIa tubulin isotype and indanocine complex, (D) βIII tubulin isotype and indanocine complex (E) βIVa tubulin isotype and indanocine complex (F) βIVb tubulin isotype and indanocine complex (G) βV tubulin isotype and indanocine complex and (H) βVI tubulin isotype and indanocine complex. (TIF) [file pone.0194934.s013.tif]
